# Oily Ascites Revealing a Spontaneous Rupture of a Mature Ovarian Cystic Teratoma

**DOI:** 10.5334/jbsr.2195

**Published:** 2020-09-11

**Authors:** Paul-Emile Colin, Louis Deprez, François Cousin

**Affiliations:** 1 Centre hospitalier universitaire de Liège, BE

**Keywords:** ovarian teratoma, teratoma rupture, chronic peritonitis, oily ascites

## Abstract

**Teaching point:** Ovarian teratoma rupture can manifest clinically as an acute or chronic syndrome, associated with specific imaging features, both characterized by intra-abdominal fatty fluid.

## Case

**Figure 1:**
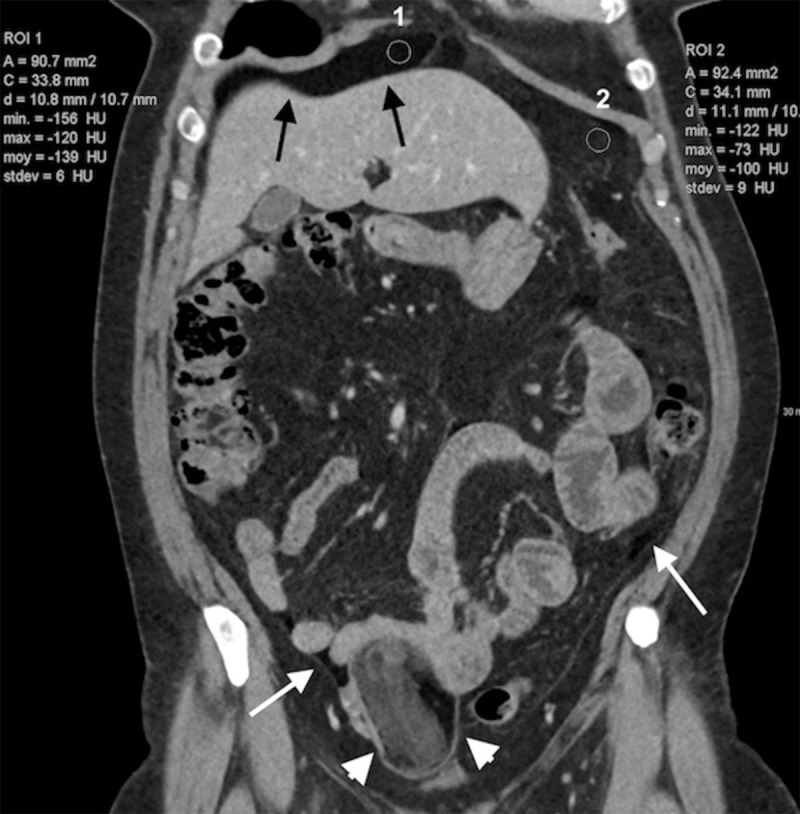


A 55-year-old female was admitted with complaints of diffuse abdominal pain and fever for three days. A computed tomography (CT) of the abdomen (Figure 1) demonstrated diffuse low-density ascites in the pelvis, left flank (white arrows) and the perihepatic space (black arrows). Region of interest (ROI) 1 in the perihepatic space revealed a mean density of –139 Hounsfield units (HU) compared to –100 HU in the ROI 2 measured in the contiguous abdominal fat. A 5-cm right ovarian mass containing fat and calcifications was also demonstrated (arrowheads). Neither biological nor imaging signs of intra-abdominal inflammation were noted. The diagnosis of intraperitoneal rupture of an ovarian mature teratoma was made and the patient was discharged.

**Figure 2:**
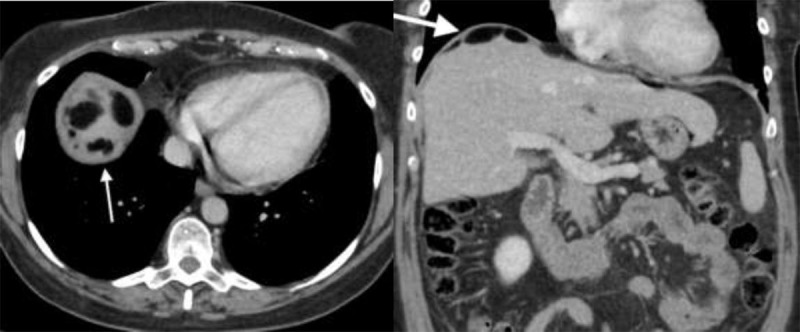


**Figure 3:**
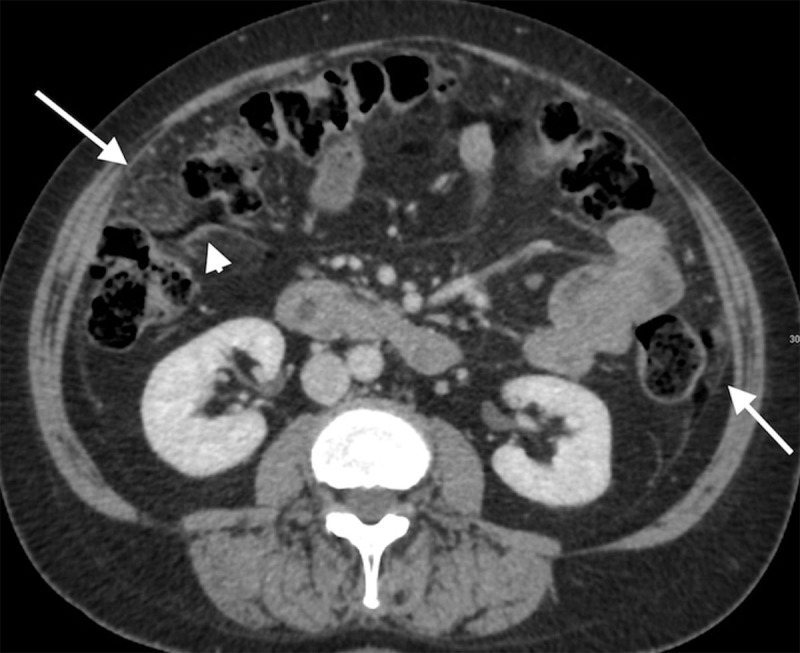


A second CT was performed three months later for persistent abdominal tenderness. It showed more organized fat zones along the mesenteric vessels, surrounding the intestine and mostly prevailing in the upper part of the abdominal cavity, under the diaphragm (Figure 2, arrows). Signs of chronic peritonitis were also visible (Figure 3), including infiltration of the intra-abdominal fat (arrows) combined with smooth thickening and enhancement of the peritoneum (arrowhead). The patient finally underwent a surgical resection confirming a ruptured right ovarian mature teratoma and diffuse oily ascites.

## Comment

Mature cystic teratomas are one of the most common ovarian benign masses. They can contain different components as mature skin, hair follicles, nails, or teeth. Fat is present in almost all lesions, although a small proportion shows minimal or no fat which leads to challenging imaging diagnoses.

Most frequent complications of teratomas are torsion and rupture, which occurs in 1–4% of the cases. Teratoma rupture is usually secondary to torsion, infection, direct trauma, or delivery. Spontaneous rupture is less frequent because of the thick capsule that typically surrounds the tumour. It induces intra-peritoneal leakage of the sebaceous content of the tumour, causing peritoneal inflammation. Two different clinical syndromes are described: acute rupture is associated with abdominal pain or heaviness with nausea and vomiting, while chronic rupture is a more indolent syndrome characterized by diffuse abdominal tenderness or discomfort secondary to chronic granulomatous peritonitis [1].

CT is the most sensitive technique to assess teratoma rupture, showing patchy hypoattenuating fatty fluid collection in the abdominal cavity, characteristically below the right hemi-diaphragm. In chronic rupture, signs of chronic peritonitis are typically found like diffuse intra-abdominal fat stranding and peritoneal thickening. These findings should not be mistaken for tuberculous or carcinomatous peritonitis.
